# Fatty acid metabolism-related enzymes in colorectal cancer metastasis: from biological function to molecular mechanism

**DOI:** 10.1038/s41420-024-02126-9

**Published:** 2024-08-05

**Authors:** Biao Li, Jing Mi, Qi Yuan

**Affiliations:** College of Life Sciences, Mudanjiang Medical University, Mudanjiang, China

**Keywords:** Transferases, Cancer metabolism

## Abstract

Colorectal cancer (CRC) is a highly aggressive and life-threatening malignancy that metastasizes in ~50% of patients, posing significant challenges to patient survival and treatment. Fatty acid (FA) metabolism regulates proliferation, immune escape, metastasis, angiogenesis, and drug resistance in CRC. FA metabolism consists of three pathways: de novo synthesis, uptake, and FA oxidation (FAO). FA metabolism-related enzymes promote CRC metastasis by regulating reactive oxygen species (ROS), matrix metalloproteinases (MMPs), angiogenesis and epithelial-mesenchymal transformation (EMT). Mechanistically, the PI3K/AKT/mTOR pathway, wnt/β-catenin pathway, and non-coding RNA signaling pathway are regulated by crosstalk of enzymes related to FA metabolism. Given the important role of FA metabolism in CRC metastasis, targeting FA metabolism-related enzymes and their signaling pathways is a potential strategy to treat CRC metastasis.

## Facts

- FA metabolism-related enzymes profoundly affect various physiological processes of CRC metastasis, and precisely targeting FA metabolism-related enzymes is a new direction of cancer therapy.
- FA metabolism-related enzymes promote CRC metastasis by regulating reactive ROS, MMPs, angiogenesis, and EMT.
- FA metabolism-related enzymes are involved in various pathways in CRC metastasis, including PI3K/AKT/mTOR pathway, wnt/β-catenin pathway, and non-coding RNA signaling pathway. An imbalance of any enzyme will affect CRC metastasis.

## Open questions

- What is the role of FA metabolism in tumor progression?
- How do FA metabolism-related enzymes play a regulatory role in colorectal cancer metastasis?
- What are the clinical treatment drugs for CRC metastasis?

## Introduction

Colorectal cancer (CRC) is the cause of the second largest number of cancer deaths globally, with incidence rates continuing to rise in developed countries [1]. By 2024, ~152,810 cases of CRC are expected to be diagnosed in the United States (7.6% of new cancer cases in the United States) and ~53,010 deaths (8.6% of cancer deaths in the United States) [2]. Unfortunately, the global incidence of CRC is expected to increase to 2.5 million cases by 2052 [3]. Recent studies have shown that the incidence and mortality of CRC not only affect the elderly but also increase in young people [4]. Multiple risk factors such as genetics, environment, and poor living habits affect the occurrence and development of CRC [5, 6].

Distant metastasis is the main cause of CRC-related death, and the liver is the most common organ affected by CRC metastasis [7]. In clinical patients, 50% of CRC eventually develop liver metastasis [8]. The second most common organ involved in the distant metastasis of CRC is the lung [9, 10]. Recent studies have shown that 10-15% of CRC develop lung metastases, and the 5-year survival rate of patients diagnosed with metastatic CRC is less than 20% [11–13].

Abnormal alterations in energy metabolism have become important markers of cancer, and fatty acid (FA) metabolism is generally enhanced at different stages of tumor progression [14]. To metastasize, tumor cells use FA metabolism to promote different steps of the metastasis cascade, from the initiation of metastasis to the promotion of cell colonization growth after metastasis [15]. FA-related metabolic and structural adaptations in tumors include changes in lipid membrane composition to invade other sites, overcoming cell death mechanisms, and promoting lipolysis and anabolism for energy and oxidative stress protection purposes [16].

Studies have shown that obesity is a risk factor for 13 types of cancer, including CRC, and tumor cells can meet their energy needs for rapid proliferation through lipid metabolism [17–19]. Lipid biology includes FA metabolism, fat and cholesterol homeostasis, which are important processes in tumor metastasis [20]. FA accumulation is more significant in metastatic tumors than in primary tumors. In lipid-rich lymph nodes, metastatic tumor cells tend to use fatty acids as the primary fuel for energy production [21]. In ovarian cancer, adipocytes promote tumor growth by supplying FA to cancer cells, which in turn promote metastasis [22]. The energy storage/production hypothesis should be the most important mechanism of tumor metastasis.

Therefore, this review focuses on recent advances in understanding the relationship between FA metabolism and CRC metastasis.

A systematic literature search of PubMed was conducted to identify articles published between January 1, 2014, and January 1, 2024, focusing on the following areas of interest: “Colorectal cancer”, “metastasis”, and “fatty acid metabolism”. Boolean operators are used to create targeted search strategies. After selecting the keywords, a search was performed in PubMed “All Fields”.

## Structure and functions of FA

FA consisting of carboxyl groups and hydrocarbon chains of varying carbon lengths and unsaturation are involved in cell signaling, regulation of immune responses, and maintenance of homeostasis in the internal environment [23–25]. FA can be divided into saturated fatty acids (SFAs) without double bonds, monounsaturated fatty acids (MUFAs) containing one double bond, and polyunsaturated fatty acids (PUFAs) containing at least two double bonds according to the number of double bonds [26]. Palmitic acid (PA) is a kind of SFAs. PA has anti-inflammatory, anti-oxidation, and immune-enhancing effects. PA induces tumor cell apoptosis, inhibits tumor cell proliferation, inhibits metastasis and invasion, enhances sensitivity to chemotherapy, and improves immune function [27]. Oleic acid is one of the most abundant MUFAs, regulating cell membrane fluidity, receptors, intracellular signaling pathways, and gene expression [28, 29]. The PUFA family includes α-linolenic acid, stearic acid, eicosapentaenoic acid, docosapentaenoic acid, and docosahexaenoic acid, which can provide energy for the body, inhibit the inflammatory response, and regulate body metabolism [30]. FA exists in various cellular structures and regulates biochemical activities of normal cells, including the generation and regulation of biofilm fluidity, as a second messenger of signaling pathways, and as a mode of energy storage [31–33]. Normal cells mainly take up exogenous FA, while tumor cells promote FA production [34]. Therefore, understanding the mechanisms by which FA regulates CRC metastasis are critical for the prevention and treatment of CRC.

## Mechanism of FA metabolism-regulating tumor metastasis

### FA metabolism regulates matrix metalloproteinases (MMPs)

**Fig. 1:**
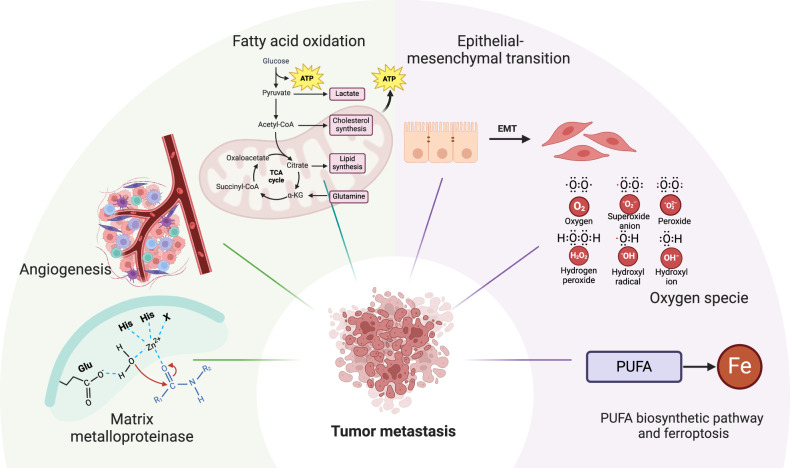
FA metabolism affects the mechanism of tumor metastasis. FA metabolism with MMPs, regulates angiogenesis, ROS, EMT, FAO effect mechanism of tumor metastasis (Created with Biorender.com).

MMPs are the proteases that remodel and degrade extracellular matrix [35]. MMPs are released by various connective tissues and cells, including fibroblasts, osteoblasts, endothelial cells, macrophages, neutrophils, and lymphocytes [36]. Multiple studies have shown that FA metabolism is involved in various pathological processes by regulating MMPs, such as promoting tumor angiogenesis, invasion, and metastasis [37]. For example, microRNA-199a-3p activated the phosphatidylinositol-3-kinase/ (PI3K)/AKT signaling pathway and upregulated the expression of MMP2 by regulating the expression of stearoyl-CoA desaturase 1 (SCD1), thereby inhibiting the metastasis of nasopharyngeal carcinoma [38]. In CRC, sterol regulatory element-binding protein 1 (SREBP1) promoted tumor cell metastasis by activating the nuclear factor-κB (NF-κB)/MMP7 axis [39] (Fig. 1).

### FA metabolism regulates angiogenesis

Angiogenesis depends on an adequate blood supply and is an important cause of tumor growth and metastasis [40]. In animal models of xenografted tumors, the tumor microenvironment is hypoxic when the diameter is greater than 2 mm. As a result, tumors need to form new blood vessels to provide oxygen and essential nutrients [41–43]. Vascular overgrowth and upregulation of pro-angiogenic factors promote tumor metastasis, the degree of which is affected by pro-angiogenic factors [44]. FA metabolism can promote tumor metastasis by regulating angiogenesis. Gao et al. demonstrated that SREBP1 overexpression promoted angiogenesis in endothelial cells, which in turn promoted the invasion and metastasis of CRC cells [45] (Fig. 1).

### FA metabolism regulates epithelial-mesenchymal transition (EMT)

EMT is a complex cellular process executed by EMT-activating transcription factors, mainly the zinc finger protein SNAI, Twist‐related protein and zinc finger E‐box‐binding homeobox families [46–48]. In solid tumors, EMT plays a role in tumorigenesis, invasion, metastasis, cancer stemness and drug resistance [49]. FA metabolism is widely confirmed to be involved in the process of EMT. Du et al. showed that downregulation of miR-130b promoted the expression of peroxisome proliferators-activated receptors gamma (PPAR-γ), which subsequently inhibited EMT in hepatocellular carcinoma [50]. Long-chain acyl-coenzyme A synthases family member 3 Gene (ACSL3) stimulated EMT of CRC cells, providing fuel for tumor cell invasion and distant metastasis [51] (Fig. 1).

### FA metabolism regulates reactive oxygen species (ROS)

ROS as a normal cell signaling molecules of biological processes [52]. ROS includes oxygen radicals, such as superoxide anion radicals (O_2_^−^) and hydroxyl radicals (OH), and non-radical oxidants, such as hydrogen peroxide (H^·^_2_O_2_) and singlet oxygen (^1^O_2_) [53]. ROS acts as signaling molecules in cancer, leading to cell growth, metastasis and angiogenesis [54]. In ovarian cancer, increased ROS production and activation of the AKT/mammalian target of rapamycin (mTOR) signaling pathway were involved in the upregulation of SREBP1 and SREBP2, which promoted cell growth and metastasis [55] (Fig. 1).

### FAO regulates tumor metastasis

FAO is a whole process involving FA activation, transporting, β oxidation and TCA cycle. FAO mainly focuses on β-oxidation, and the important site of β-oxidation is mitochondria, which generates acetyl-CoA through a series of reactions and enters TCA cycle coupled oxidative phosphorylation to produce ATP and reduced Coenzyme II, providing corresponding energy and reducing power for cell growth. CPT1, located in the outer membrane of mitochondria, catalyzes the esterification of long-chain acyl groups with carnitine and is a key rate-limiting step of FAO, mediating cellular FAO [56]. Studies have shown that significantly elevated expression of transcriptional coactivator yes-associated protein (YAP) can mediate the production of FAO in tumor cells during lymph node metastasis, and this metabolism contributes to the metastasis of tumor cells [20]. Nuclear Receptor Nur77 promotes FAO in glucose starvation and promotes melanoma metastasis by protecting the key FAO enzyme TPβ from oxidative inactivation [57]. FAO helps AMPK regulate NADPH homeostasis in cancer cells, and FA β-oxidation promotes cancer cell survival under energy stress, thus promoting CRC metastasis [58, 59] (Fig. 1).

### PUFA biosynthetic pathway and ferroptosis

The ferroptosis was first proposed by Dixon et al. in 2012. Ferroptosis refers to the type of iron-dependent regulatory cell death induced by lipid peroxidation in cell membranes [60]. The link between PUFA and ferroptosis is that PUFAs are particularly prone to oxidation, which leads to the formation of lipid peroxides, resulting in membrane rupture and eventual cell death. Interestingly, the balance between omega-3 polyunsaturated fatty acids and omega-6 polyunsaturated fatty acids could also play a role in ferroptosis. Omega-3 polyunsaturated fatty acids are more prone to oxidation and may promote ferroptosis, whereas omega-6 polyunsaturated fatty acids can be converted into proinflammatory molecules that prevent ferroptosis by activating antioxidant pathways [61]. ELOVL2, ELOVL5, FADS1, and FADS2, these enzymes play an important role in the PUFA synthesis pathway and also play an important role in ferroptosis [62, 63]. The decreased expression of ELOVL5 and FADS1 in gastric cancer and CRC cells suggests that these two enzymes can be used as predictive markers for ferroptosis-mediated cancer therapy [62] (Fig. 1).

## The role of FA metabolism-related enzymes in CRC metastasis

In recent years, the role of FA metabolism-related enzymes in CRC metastasis has been extensively studied, and different enzymes promote metastasis through unique mechanisms involving the MMP, EMT, angiogenesis, and ATP production (Table 1). The molecular mechanism of FA metabolism-related enzymes regulating CRC metastasis will be elucidated in this review.

**Table 1:** Expression, discovery, and influence of fatty acid metabolism-related enzymes in CRC metastasis.

Enzymes	Expression	Feature	Influence	Reference
SREBP1	Upregulate	Angiogenesis	Tumor invasion	[45]
ELOVL5	Downregulate	PUFA synthesis	Prediction marker	[62]
ACLY	Upregulate	Drug resistance	Tumor metastasis	[71]
ACSL3	Upregulate	EMT	Tumor metastasis	[51]
FASN	Upregulate	ATP production	Tumor metastasis	[82, 83]
SCD1	Upregulate	ATP production	Tumor metastasis	[82]
CD36	Upregulate	MMP28	Tumor metastasis	[93]
FABP5	Downregulate	IL-8	Tumor metastasis	[101]
PPAR	Upregulate	FAO rate	Tumor metastasis	[105]
CPT1A	Downregulate	FAO activation	Tumor metastasis	[109]
CPT1C	Upregulate	FAO rate	Tumor metastasis	[112]

### The role of FA synthesis-related enzymes in CRC metastasis

#### SREBP

SREBF contains two homologous genes and three isoforms: SREBP1a and SREBP1c, which are encoded by the SREBF1 gene, and SREBP2, which is encoded by the SREBF2 gene [64]. SREBF1 mainly regulates the synthesis of FA and triglyceride (TG), while lipoprotein uptake and de novo cholesterol synthesis are mainly regulated by SREBF2 [65]. In the process of FA metabolism, the expression of various FA metabolism-related genes is regulated by SREBP1, including acetyl-CoA carboxylase (ACC), fatty acid synthetase (FASN), and SCD1 [66]. Multiple studies have shown that SREBP1 promotes CRC metastasis by regulating downstream FA metabolism-related enzymes [45]. In addition, SREBP1 not only regulates the synthesis of various lipids in cells but also plays a key role in the occurrence and development of malignant tumors. SREBP1 promotes CRC metastasis by influencing ROS, increasing MMPs expression, and angiogenesis. Gao et al. found that overexpression of SREBP1 in HT29 cells promoted endothelial cell angiogenesis, increased ROS and MMP7 expression, and further promoted the invasion and metastasis of CRC cells [45]. SREBP1 also promoted the invasion of CRC cells by increasing ROS to activate NF-κB/MMP7 axis [39]. Therefore, the development of targeted drugs against SREBP1 may be a new direction for the treatment of CRC metastasis.

#### ATP citrate lyase (ACLY)

The first key enzyme in de novo lipogenesis is ACLY, which is an important bridge connecting cellular glucose and lipid metabolism [67]. Citrate enters the cytoplasm from mitochondria and is catalyzed by ACLY to generate Ac-CoA, which in turn is an important raw material for FA synthesis. Therefore, changes in energy metabolism in tumor cells, increased glucose uptake and increased glycolytic flux accelerate the increase in citrate yield, ultimately promoting cellular FA synthesis [68, 69]. ACLY is related to the migration and invasion ability of CRC cells by Pearson correlation analysis [70]. Abnormal expression of ACLY contributes to enhancing the drug resistance of metastatic CRC [71]. In addition, Qiao et al. demonstrated that homeobox A13 (HOXA13) promoted CRC metastasis by transactivating ACLY and insulin-like growth factor receptor (IGF-1R) [72].

#### ACSL

ACSL regulates CRC metastasis mainly by affecting EMT. In mammals, the ACSL family comprises five members, ACSL1 and ACSL3-6 [73]. In the liver, heart, adipose, and muscle, ACSL1 is widely expressed [74]. It acts as a key enzyme in FA uptake, FAO, and TG synthesis [73]. ACSL3 promotes the production of ATP and reduces nicotinamide adenine dinucleotide phosphate (NADPH), stimulate EMT and metastasis in CRC cells [51]. Current studies have shown that the downregulation of ACSL can predict the prognosis of CRC with metastasis at an early stage, and targeting ACSL3 to affect EMT provides new targets and ideas for clinical treatment.

#### Acetyl-CoA carboxylase (ACC)

ACC (including ACC1 and ACC2), a key enzyme in FA synthesis, regulates the adipogenesis, metastasis, and apoptosis of CRC cells. The first rate-limiting enzyme in the de novo FA synthesis pathway is ACC1, which is located in the cytoplasm [75]. ACC2 produces Malonyl-CoA during de novo FA synthesis [76]. Malonyl-CoA also plays an important role in FA catabolism as a direct substrate for FA synthesis [68]. It has been widely demonstrated that the role of AMP-activated protein kinase (AMPK) signaling in the growth, proliferation, angiogenesis, metastasis, and invasion of CRC. ACC is a target molecule of AMPK, and phosphorylation of AMPK regulates ACC activity [77]. On the other hand, regenerating islet-derived 4 (REG4) induces the degradation of ACC1 or the ACLY proteasome, while lymph node and distant metastasis, and the tumor-lymph node metastasis stage are negatively correlated with REG4 overexpression[78].

#### FASN

FASN regulates CRC metastasis mainly by affecting ATP production. As a key enzyme in FA synthesis, FASN is significantly elevated in cancer associated fibroblasts and plays a key role in the growth and survival of lipogenic phenotype tumors [79]. FASN is a large multienzyme complex with a monomeric protein size of ~270 kDa [80]. There are two subtypes of FASN (FASN1 and FASN2), with FASN1 found in fungi and animals and FASN2 found in prokaryotes and plants. FASN converts excess carbohydrates into FA, which is esterified by other enzymes into stored lipids that provide energy through oxidation when needed by the body [81]. Clinical studies have shown that FASN is significantly expressed in CRC [49]. FSCN1 knockdown reduced the expression of FASN and SCD1, SFA was converted to MUFA via SCD1, and FASN chains were elongated to increase the production of ATP, thereby promoting CRC metastasis [82, 83].

#### SCD

SCD promotes ATP production, which provides the necessary conditions for rapid tumor growth and metastasis initiation. SCD is a central lipogenic enzyme that catalyzes the synthesis of MUFAs from SFA, palmitate, and stearate [84]. To date, a total of four mouse SCD subtypes (1 to 4) and two human SCD subtypes (1 and 5) have been identified. Bioinformatics analysis revealed that high expression of SCD1 was associated with invasive and metastatic phenotypes of CRC [85, 86]. Ferroptosis is critical for CRC progression due to the redox imbalance of tumor cells, characterized by lipid peroxidation. Studies have shown that inhibition of SCD1 partially eliminates the resistance of Nodal growth differentiation factor (Nodal) overexpressing cells to ferroptosis, thus promoting the survival and metastasis of CRC cells [87]. Nodal overexpression induces MUFA synthesis and increases the level of unsaturated lipids. Mechanistically, the transcription of SCD1 is upregulated by Smad 2/3 signaling when Nodal is overexpressed [87]. In addition, SCD1 converts SFAs into MUFAs, and its chain is lengthened to increase ATP production, thereby promoting CRC metastasis [82]. In conclusion, targeting SCD1 may become a novel therapeutic strategy for the treatment of CRC metastasis.

### The role of FA uptake-related enzymes in CRC metastasis

#### CD36

**Fig. 2:**
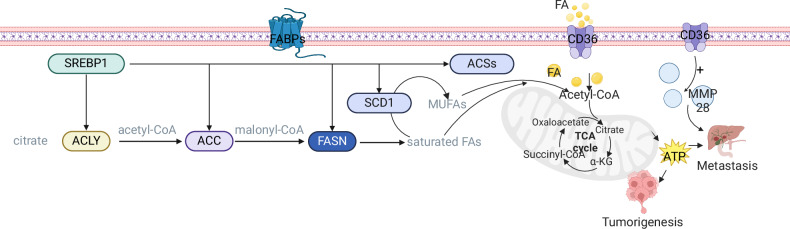
FA metabolism in CRC. SREBP1 regulates the expression of ACLY, ACC, FASN, SCD1, and ACS at the transcriptional level. CD36, which allows cells to absorb lipids from the extracellular microenvironment and promotes the production of ATP, can also affect MMP28, thereby affecting tumor development and metastasis (Created with Biorender.com).

CD36 is a member of scavenger receptor family B and is expressed on the surface of various cells, such as adipocytes, hepatocytes, and cardiomyocytes [88]. CD36 is involved in tumor pathogenesis by regulating the mitochondrial gene PPAR [89]. High expression of CD36 in CRC tissues is associated with malignant transformation and predicts poor survival of CRC based on bioinformatics analysis [90]. CD36 allows the uptake of lipids from the extracellular microenvironment by cells and promotes ATP production, which stimulates tumor progression and metastasis [91, 92]. On the other hand, CD36 plays an important regulatory role in CRC metastasis by upregulating MMP28 [93] (Fig. 2).

#### Fatty acid binding protein (FABP)

FABP is a family of multifunctional proteins involved in FA metabolism. FABP is classified into 12 subtypes according to different tissue sources [94]. In lipid metabolism, long-chain FAs bind to FABP to improve FA solubility and facilitate FA transport to cell or mitochondrial membranes [95, 96]. At present, the expression level of FABP in CRC is still inconclusive. Based on ONCOMINE and GEPIA2 analyses, Prayugo et al. found that the expression of the FABP1 and FABP6 genes was significantly increased in CRC, and different tumor stages of CRC were related to the expression levels of FABP3 and FABP4 mRNA [97, 98]. Consistent with Prayugo’s analysis results, recent studies have shown that FABP4 is more expressed in tumor node metastasis stages I-II than III-IV and is involved in metabolic reprogramming, tumor differentiation, and metastasis [99, 100]. However, FABP1 was downregulated in CRC tissues according to the single cell transcriptome analysis of epithelial cells from 272 CRC and 160 normal epithelial cells [97]. This finding further suggested that FABP may play dual roles in CRC progression. Mechanistic studies have shown that FABP5 expression is upregulated in metastatic CRC cells by continuously promoting DNA demethylation and activation of the NF-κB pathway, which in turn regulates NF-κB activity through IL-8 production [101]. Further study on the specific mechanism of FABP in CRC will help to provide new strategies for targeted therapy in CRC.

### The role of FAO-related enzymes in CRC metastasis

#### PPAR

PPAR has three isoforms, α, β and γ. PPAR-α is highly expressed in the liver and is involved in physiological activities such as energy metabolism, oxidative stress, and inflammation. PPAR-β plays a role in inhibiting the inflammatory response. PPAR-γ is widely expressed in various adipose tissues and participates in lipid metabolism [102, 103]. In mice, DNMT1-mediated Cdkn1a (P21) methylation and PRMT6-mediated increases in Cdkn1b (P27) methylation lead to PPAR-α reduced and promote colon tumorigenesis and growth [104]. In CRC, the protein tyrosine phosphatase receptor type O gene (PTPRO) promotes the expression of FAO enzymes by upregulating PPAR-α, thereby promoting tumor metastasis [105]. The Nanog promoter in CRC cells directly binds to PPAR-β to induce Nanog expression, which in turn induces colon cancer stem cell (CSC) expansion and CRC liver metastasis [106].

#### Carnitine palmitoyl transferase (CPT)

CPT is a key FAO enzyme that is present in the inner membrane of mitochondria. The CPT family consists of CPT1 and CPT2. CPT1 is located outside the mitochondrial membrane and includes three tissue-specific isoforms. CPT2 is a widely distributed protein located in the inner mitochondrial membrane [107, 108]. Long-chain FA must be converted to acyl-carnitines before entering the mitochondrial matrix for oxidation [108]. Malonyl-CoA (a component of ACC) blocks the activity of CPT1, FAO is accelerated when ACC is inhibited. Previous studies have shown that phosphorylation of ACC reduces tumor cell proliferation by enhancing FA degradation [83, 109]. The expression level of CPT1A was found to be reduced in peritoneal metastatic CRC [110]. In addition, FAO activation mediated by CPT1A and CPT1C increases the metastatic capacity of CRC [111, 112].

## Signaling pathways regulating FA metabolism in CRC metastasis

### PI3K/AKT/mTOR signaling pathway

**Fig. 3:**
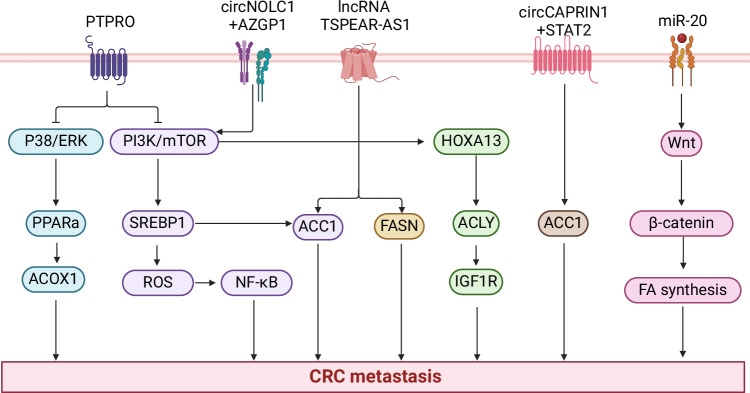
Signaling pathways regulating with FA metabolism in CRC metastasis. PI3K/AKT/mTOR signaling pathway, Wnt/β-catenin signaling pathway, MAPK signaling pathway, and Noncoding RNA regulate CRC metastasis in a variety of ways (Created with Biorender.com).

The PI3K/AKT/mTOR signaling pathway promotes tumor cell proliferation, metabolism, and angiogenesis [113]. In recent years, it has been found that the PI3K/AKT/mTOR signaling pathway promotes CRC metastasis by regulating FA synthesis-related enzymes. Insulin-like growth factor 1 (IGF1) upregulates HOXA13 expression via the PI3K/AKT signaling pathway. HOXA13 promotes CRC metastasis through transactivation of ACLY and IGF1R [72]. Silencing of PTPRO enhances the expression of SREBP1 and its target lipogenic enzyme ACC1 by activating the AKT/mTOR signaling pathway, thus promoting adipogenesis, cell growth, and liver metastasis [105]. In addition, FA metabolism reversely activates the PI3K/AKT/mTOR signaling pathway, forming a loop of positive feedback. FABP4 overexpression regulates the increase of FAs and activates AKT pathway and EMT, thereby promoting the migration and invasion of CRC cells [114] (Fig. 3).

### Wingless (Wnt)/β-catenin signaling pathway

Wnt signaling pathway includes nonclassical pathway independent of β-catenin and classical pathway known as Wnt/β-catenin pathways [115]. The Wnt/β-catenin signaling pathway is a conserved signal transduction axis involved in various physiological processes such as proliferation, differentiation, apoptosis, migration, invasion, and tissue homeostasis [116]. β-catenin is a key mediator of Wnt signal transduction and is present in multiple subcellular locations, including adhesive junctions, where it helps stabilize cell-to-cell contact. Its levels in the cytoplasm are controlled by processes that regulate protein stability and are involved in transcriptional regulation and chromatin interactions in the nucleus [117]. Hai et al. found that FASN expression was positively correlated with Wnt signaling-related gene expression, and genetic perturbation indicated that FASN knockdown inhibited cell migration and invasion of CRC cell lines [118]. In addition, miR-20 activated the Wnt/β-catenin signaling pathway and upregulated FA synthesis, ultimately promoting the proliferation and migration of metabolic CRC cells [119] (Fig. 3).

### Noncoding RNA

Noncoding RNA is a group of functional RNA that cannot be transcribed into proteins but are involved in various biological processes that regulate gene transcription and translation. Noncoding RNA accounts for 98% of the whole genome transcripts, including microRNA (miRNA), long-stranded non-coding RNA (lncRNA), small interfering RNA (siRNA), and circular RNA (circRNA) [120]. The expression level of miRNA-27a-3p is negatively correlated with CD36 in CRC metastatic [79, 121]. Peng et al. demonstrated that FASN and ACC1 were regulated by lncRNA-TSPEAR-AS1 to promote CRC progression [122] (Fig. 3). circCAPRIN1 interacts with signal transducer and activator of transcription 2 (STAT2) to increase the expression of ACC1 and promote tumor progression and lipid synthesis of CRC cells [123] (Fig. 3). In CRC liver metastasis, circNOLC1 interacts with Zinc-α2-glycoprotein 1 (AZGP1) to activate mTOR/SREBP1 signal transduction and the oxidative pentose phosphate pathway [124] (Fig. 3).

### MAPK signaling pathway

MAPK signaling pathway is a classical intracellular signal transduction system that controls a variety of cellular processes, such as cell differentiation, proliferation, and apoptosis [125]. MAPK consists of three major families: extracellular signal-regulated kinase 1/2 (ERK1/2), c-Jun N-terminal kinase (JNK), and p38. These families play an important role in CRC carcinogenic effects [126]. PTPRO attenuation activates P38/ERK signaling pathway, resulting in decreased expression of PPARα and ACOX1, thereby reducing the rate of FAO and promoting CRC metastasis [105]. RPL21 and lysosomal associated membrane protein 3 (LAMP3) promote CRC cell metastasis by activating the FAK/paxillin/ERK signaling pathway, thereby increasing the formation of immature FA [127] (Fig. 3).

## Therapeutic agents for FA metabolism in CRC metastasis

Echinoside is a major phenylethanol glycoside structural compound identified in Huangzu granules that inhibits the invasion and metastasis of CRC and prolongs the disease-free survival of patients [128]. Pterostilbene (PTS) inhibits cell migration by downregulating FABP5 gene expression thus alleviating obesity-induced CRC metastasis [129]. Cerulenin is a natural FA synthase inhibitor that can prevent and delay CRC liver metastasis [119]. Inulin, cellulose, and their mixtures inhibit CRC metastasis by regulating the intestinal microbiota, producing short-chain fatty acids, and inhibiting EMT [130]. Mangiferin inhibits tumor growth, metastasis, and angiogenesis by targeting mitochondrial redox enzymes and FAO metabolic signaling pathways [131]. Resveratrol is a polyphenolic compound with antioxidant, anti-inflammatory, apoptosis-inducing, and anti-angiogenic properties. It plays an important role in the treatment of CRC [132, 133] (Table 2).

**Table 2:** Therapeutic drugs used in clinical treatment and their mechanisms of action.

Drug name	Drug properties	Reference
Echinoside	Down-regulated PI3K/AKT signaling and EMT	[128]
Pterostilbene	Down-regulated FABP5 gene expression	[129]
Cerulenin	FA synthetase inhibitors	[119]
Inulin	Regulate intestinal flora and produce short-chain fatty acids	[130]
Mangiferin	Targeting mitochondrial oxidoreductase and FAO metabolism	[131]
Resveratrol	Antioxidant, anti-inflammatory, apoptosis-inducing and anti-vascular	[132, 133]

## Conclusion

In summary, FA metabolism is an important trigger of CRC metastasis. FA metabolism-related enzymes (SREBP1, ACLY, ACSL, ACC, FASN, SCD1, etc.) regulate ROS, EMT, MMPs, and angiogenesis in tumor cells, thereby promoting CRC metastasis. Having learned the important role of FA metabolism in CRC metastasis, an understanding of its regulatory mechanisms will allow researchers the opportunity to develop new therapeutic approaches. These may include :(1) targeting FA metabolizing enzymes and inhibiting tumor metastasis. (2) indirectly regulate FA metabolism by targeting the regulators and signaling pathways of FA metabolism.

With the rapid development of the medical and health industry today, the speed and depth of medical research have reached an unprecedented level. In-depth understanding of the mechanism of FA metabolism depends on large-scale clinical data, but in reality, the difficulty factor of sample acquisition is large, and the research results are stagnant due to the insufficient sample size, which cannot meet our current further research on FA metabolism.

At present, the types of targeted inhibitors of FA metabolic-related enzymes in the market are limited, which limits our in-depth understanding of FA metabolic-related diseases and the innovation of treatment methods. In order to fill this historical gap, it is particularly urgent to expand the research on targeted inhibitors. This includes not only the discovery of new inhibitors, but also the optimization and modification of existing inhibitors to improve their selectivity and effectiveness.

Cancer metastasis is a complex multi-step process, involving various links such as cell invasion, migration, angiogenesis, and infiltration and integration in a new environment. Which link has a potential connection with FA metabolism-related enzymes is a great challenge for current research work. Through the cellular and molecular biology research and large-scale genome and transcriptome analysis, can further understand the stage of cancer metastasis and the connection between the FA metabolism-related enzymes.
